# High non-compliance rate with anti-tuberculosis treatment: a need to shift facility-based directly observed therapy short course (DOTS) to community mobile outreach team supervision in Saudi Arabia

**DOI:** 10.1186/s12889-019-7520-8

**Published:** 2019-08-27

**Authors:** Abdullah Jaber AlSahafi, Hassan Bin Usman Shah, Mashal Mesfer AlSayali, Najlaa Mandoura, Mohammed Assiri, Emad Lafi Almohammadi, Alaa Khalawi, Abdullah AlGarni, Maimona Kamal Filemban, Adel Khaled AlOtaibe, Abdulaziz W. A. AlFaifi, Fatima AlGarni

**Affiliations:** 1 General Directorate of Health Affairs for Public Health Division, Jeddah, Saudi Arabia; 2 Research Department, Directorate of Health Affairs for Public Health Division, Jeddah, Saudi Arabia; 3 General Directorate of Health Affairs, Jeddah, Saudi Arabia; 4 TB DOTS program, Department of Public Health, Jeddah, Saudi Arabia; 5 Infection control Department of Ministry of Health, Jeddah, Saudi Arabia; 6 TB control program, Department of Public Health, Jeddah, Saudi Arabia; 7 Communicable Diseases Department- Public Health, Jeddah, Saudi Arabia

**Keywords:** Compliance, Default rate, Directly-observed therapy short course (DOTS), Mobile outreach teams, Treatment outcome, Tuberculosis

## Abstract

**Background:**

Tuberculosis (TB) remains a major global public health problem in many developing countries including Kingdom of Saudi Arabia (KSA). Patient compliance with anti-tuberculosis treatment is a determining factor in controlling the spread of TB. This study compares the default rate and the perception of their treatment among TB patients being treated by means of a community mobile outreach approach, with those of patients being treated by means of a facility-based Directly Observed Treatment Short course (DOTS) in the Jeddah region of Saudi Arabia.

**Methods:**

A comparative cross-sectional study of 200 TB patients who presented at the Madain Alfahd Primary Health Care Center (PHCC) Jeddah, between January 2018 and November 2018 was undertaken. In one group, randomly assigned patients were served by mobile outreach teams who administered oral anti-TB treatment under the DOTS regime. In the other group, the patients were treated by means of the traditional facility-based DOTS treatment. A questionnaire measuring patient attitudes and understanding of the disease and their treatment modes was completed by patients at the beginning of their treatment, and again after 3 months. The results were analysed by means of independent and Paired T Tests, along with chi square analysis.

**Results:**

We found that the overall default rate among those patients served by our mobile outreach team was only 3%, compared with a 22% default rate among non-mobile team treated patients (*p* = < 0.001). A major change in the attitude and understanding scores of patients was noted in both groups after 3 months. A significant difference was also noted in the mean compliance scores (mobile team served =58.43 and facility-based =55.55, *p* < 0.001) after 3 months of treatment.

**Conclusion:**

Our study indicated that treatment by means of our mobile outreach DOTS can offer an effective strategy for the treatment of TB patients. A reduced patient default rate and a better understanding of the disease and its treatment confirmed a positive impact of mobile outreach teams on these patients. Treating TB patients by means of mobile outreach teams can thus be recommended as a means for the cure and prevention of the further spread of the disease.

## Background

Tuberculosis (TB) is a curable and preventable disease, yet it remains a major public health concern in many parts of the world. Despite improved diagnosis, treatment, and prevention programs, it is responsible for the deaths of more young and middle-aged adults than any other infectious disease [1]. According to the World Health Organization (WHO) the incidence of TB worldwide is around 10.4 million cases each year, with 1.5 million deaths occurring annually [1–3]. More than 90% of these deaths are reported from low- and middle-income countries, and this figure is probably still an underestimate of the actual numbers of fatalities caused by the disease [3, 4]. Under-reporting of actual numbers of cases, and the issue of undiagnosed cases are most probably the major reasons behind this suspected miscalculation [4, 5].

Successful TB management requires the administration of anti-TB drugs for at least 6 months [6]. The long duration of the treatment, which needs to continue even after apparent clinical recovery, is one of the main reasons for patient non-completion of their courses of treatment [5–7]. Among other explanations for patients’ non-completion of recommended treatment are the fear of side effects, social stigmatization, illiteracy and inadequate knowledge on the part of patients and their families about the possible serious outcomes of the disease (like relapse, drug resistance, prolonged hospitalization or even death) [8–11]. Defaulting on anti-TB medication is a major obstacle to its global and local control. Several recent reports have highlighted the worldwide reemergence of TB, and more specifically its effect in developing countries, of which Saudi Arabia is one [12]. In order to address the issue of patient non-compliance with treatment, the WHO launched a ‘Directly Observed Treatment Short Course (DOTS)’ strategy in 1993 [5, 13]. In accordance with this strategy, a treatment observer whose key task is to ensure that the patient is taking their medicine, is assigned to each patient. With DOTS, the patient meets with the treatment observer (healthcare worker, or family member or any other appropriate person) either every day or several times a week. In our study, the DOTS close supervision of patients to make sure they took their medicines, was applied both in the facility-based treatment regime and the mobile outreach program. In practice, these DOTS therapeutic regimens have proved to be highly effective in reducing the treatment failure rate associated with patient non-compliance [4, 5].

According to the WHO, the Kingdom of Saudi Arabia (KSA) has a low to middle incidence of TB. Although the incidence rate of TB has decreased here from 18 in 100,000 in the early 2000’s, to around 12 in 100,000 of inhabitants in 2018, still it is high [13–16]. Among the major reasons for its re-emergence in the Kingdom is the influx of foreign workers seeking to fill the many jobs in the country, available as a result of its vast economic expansion [15]. At any one time, these non-Saudi natives account for more than 12 million of the total population of around 33 million [14]. Immigrants to the KSA annually number around 600,000 [14]. Many of these immigrants tend to originate from TB-endemic countries in Asia and Africa [14–16]. Recent studies have highlighted that, in addition to the legal workforce, a sizeable number of the illegal immigrants in the Kingdom are not only carrying the bulk of the drug resistant strains of TB, but, once diagnosed, make up a large proportion of treatment defaulters [16–19]. They have been identified as having considerably higher TB incidence rates than those of the local population (52.2–65.6%) in different parts of the country [16–18]. Regional differences in TB incidence rates have been clearly demonstrated in several studies [18]. In addition, as the KSA is a center for pilgrimages to the Holy Mosques, millions of pilgrims, often coming from high TB-endemic Asian and African regions, travel here to perform the Hajj or Umrah throughout the year. In 2018 alone, more than 2.3 million pilgrims performed religious rituals in the KSA, and more than 1.7 million of them were foreign nationals [14]. Such mass gatherings, often involving physical activity of moderate to vigorous intensity, added to the immuno-compromised status of many of the older pilgrims, and can lead to the transmission of infectious diseases, TB being one of them [16–20]. The western region, where the majority of immigrants and pilgrims are to be found, showed clear evidence of higher incidences of TB [16, 18].

More than 30 years ago, the Ministry of Health (MOH) in the KSA launched the National TB Control and Prevention Program (NTCPP). In 1999 the WHO DOTS strategy was formally adopted [16, 18]. The literature shows that even after three decades of the NTCPP and DOTS programs, TB treatment coverage is only around 87% of patients, and the treatment success rate is only 62% [5, 18, 20]. These figures are far lower than the WHO targets of 85% treatment success rates [3, 21]. Consequently, at this juncture, the Saudi MOH is taking the challenge of meeting the WHO targets for achieving and sustaining a high cure success rate very seriously.

The literature has identified patient non-compliance and interruption in drug regimens as the main obstacles to achieving cure targets and thus preventing the spread of the disease. Human factors such as interpersonal conflicts between patients and health care providers have also been seen as having a role to play [5, 18, 22]. The ultimate success of any treatment for TB depends on patient compliance with the prescribed anti-tuberculosis treatment. Even if the patient pays regular visits to clinics for DOTS as part of the National anti-TB program, health professionals and sometimes even the treatment observer are never entirely sure whether the patient is actually taking the medicine or not [4, 23–25]. Studies have shown that patients tend to misinform and lie to even the treatment observer [23, 24]. To avoid this ambiguity, and to comply with the prescribed treatment, this year the NTCPP introduced the new concept of Community Mobile Outreach Teams.

The idea behind these mobile teams is that the anti-TB drugs are administered under the supervision of health care professionals on the patient’s doorstep. Administering medicines in this way ensures better compliance with a treatment regime because it easily prevents default and will consequently improve the patient’s treatment outcome (i.e. success rate) [5]. The underlying reason for this approach is the theory that patient default can be a product of limitations on the part of both patient and provider.

Having observed the ostensible success of this outreach program in other KSA cities, notably in the capital and other Eastern cities, where a 92% treatment success rate was reported, it was decided to replicate it in Jeddah as well [5]. No study had been previously undertaken in our region to evaluate patient compliance and cure after the administration of anti-TB drugs by means of this new method. The primary objective of our consequent study was to determine what the level of patient compliance and satisfaction with their treatment when implemented by these Community Mobile Outreach Teams was when compared with those of the patients following the traditional facility-based DOTS treatment in our region of Jeddah. Moreover, our intention was also to identify and compare patients’ attitudes regarding the reasons for or against non-compliance both before and after 3 months of treatment on either regime.

## Methods

### Study type

A comparative cross-sectional study of TB patients was conducted in Jeddah, KSA from January 1st, 2018 to November 1st, 2018.

### Participants and selection

Our subjects were TB patients who had been diagnosed during the previous 2 months on visiting an MOH clinic, in this case the PHCC Madain Alfahd. We enrolled both pulmonary or extra pulmonary cases. We followed these TB patients for up to 6 months of their anti-TB treatment course in order to ascertain the outcome variables. As the study was conducted by the MOH, the recommended anti-tuberculosis drugs were given free of charge to all patients.

### DOTS versus community mobile outreach

Patients undergoing TB management under the DOTS initiative were assessed, and divided into two groups. One group had their medication administered at the place of their convenience by members of our mobile outreach teams, while the other group of patients had to go to the health facility (PHCC in this case) to get their medicines (the traditional facility-based treatment under the DOTS recommendation). A treatment observer was assigned to each patient. This person could be either a healthcare professional, a family member or a notable member of the community, and was ultimately responsible for dose administration. In addition, the patient had to return the used medicine bottles in order to get their next dose. The mobile teams consisted of health care professionals, including nurses, supervised by doctors. Thrice-weekly visits by the mobile team members were scheduled, during which they ensured that the anti-TB medicines were being taken by patients at the place and time of their choice. Frequent surprise visits by supervisors were also planned to check on the mobile outreach team service delivery. Treatment default - defined according to the WHO guidelines as an interruption of TB treatment for two or more consecutive months during the intended treatment period - was carefully monitored [2, 3, 26].

The primary goal was to measure overall patient compliance with the DOTS program at the end of 6 months and to identify the reasons for any non-compliance. In addition to measuring compliance rates, patient acceptability of and satisfaction with the method of intervention were also assessed. To evaluate these, data was collected in two phases, using the same validated questionnaire. The first phase was before the patients were allotted to their treatment groups and the second was 3 months after the anti-TB treatment had commenced. In addition, physical and sputum examinations for Acid Fast Bacilli (AFB) were carried out on patients at two, four and 6 months of treatment.

The sample of patients was selected using systematic sampling with a random start approach. Eligible participants were adults aged 18 or over who met the eligibility criteria for anti-TB therapy according to the WHO recommendations [26]. We excluded newly diagnosed cases who refused the DOTS mobile outreach team-type intervention and visiting patients (mostly pilgrims) from other countries/cities. We also excluded HIV positive TB cases from our study, as the literature indicates an incidence of higher default rates in these patients, which could have been a source of potential bias [5, 27].

To estimate the current compliance with DOTS among TB patients, we calculated the sample size using the EpiTools online sample size calculator (http://epitools.ausvet.com.au/content.php?page=SampleSize). According to the figures taken from the Infection Control Department of the Directorate of Health Affairs, part of the MOH, the incidence of those completing their six-month anti-TB course during the previous year was around 82% [17]. Using these figures, and keeping the level of significance at 95%, the desired precision at 0.05 and the TB-patient population for Jeddah at 1500, the calculated sample size was 198. After obtaining patient-informed consent and ensuring confidentiality of data, a total of 200 (100 in each group) TB patients were randomly assigned to one of the two treatment groups to receive either one or other of the two management approaches under the DOTS regimen. Ethical approval for the study was granted by the Research Ethics Board of the Ministry of Health and Directorate of Health Affairs Jeddah (H-02-J-002-00877).

### Questionnaire, validity and reliability

The validated questionnaire was adapted after a careful review of the literature and translated into Arabic [1, 28–31]. The translation was performed by two bilingual professional translators who understood the content. The translated questionnaire was then translated back into English by two other bilingual translators and compared to its original version. This procedure ensured clarity and comprehensibility of items. Any discrepancies in the comparison were discussed, and minor adjustments were made. To test the questionnaire’s content validity, the questionnaire was then once again revised by two family medicine consultants and three community medicine consultants. A pilot study was conducted to test the wording and reliability of the questionnaire but its results have not been included in those of our study. Our questionnaire eventually consisted of two parts. The first part concerned the patient’s personal and demographic information, the second part included questions regarding their views on the reasons for non-compliance. This component consisted of 14 questions. The response to each question was elicited in a 5-point Likert response format (strongly agree to strongly disagree). The range of scores for each subscale was from one (1) to five (5) with a median of 2.5, and the overall maximum score was 70. Scores of more than the mean value were considered good scores, i.e., they reflected a better understanding of the necessity for compliance with anti-TB treatment. The statements on this questionnaire were not all worded in the same direction, so some items (i.e. 1,2,3,5,7,8,9,10,13) were adjusted for their scored value. In addition, the patients served by the mobile teams were also asked for their opinions on the competence of the teams and the method of treatment, using a validated questionnaire [29, 30]. As a standard protocol from the Ministry of Health, a health education program about TB, its treatment and the consequences of not completing the recommended course was presented to both groups during initial visits with the aid of flip charts.

### Statistical analysis

The baseline characteristics of the patients in the two treatment groups were reported using frequency distributions and descriptive statistics. The Kaplan-Meier curve was also drawn at the end of each month in order to make an overall estimate of how many patients were still continuing treatment in each of the two management groups. The difference in the patterns of default over time between these groups was studied using the log rank test. A chi square test was used to measure any significant difference between the two groups. Paired and independent T tests were used to compare the mean difference. *P* value < 0.05 was considered significant. All analyses were done using IBM SPSS Statistics for Windows, version 23.0 (IBM Corp., Armonk, NY, USA).

## Results

Our study included 200 participants with a mean age of 34.85 ± 12.94 years. In our study sample, the majority of participants were male (*n* = 133, 66.5%). Only around 29.0% (*n* = 58) were Saudi nationals, with an overall majority of non-Saudi patients i.e. 71% (*n* = 142). Saudi female patients made up 21% (*n* = 14) of subjects as compared to 79% (*n* = 52) females of other nationalities. Pulmonary TB was the most prominent TB type (*n* = 161, 80%) with a few additional cases of extra-pulmonary TB (*n* = 39, 20%). No significant difference was noted between default rates and gender (*p* = 0.306), educational status (*p* = 0.092) or TB type (*p* = 0.946). However, a significant difference in the default rate was noted between those subjects in the group treated by the mobile team and those in the facility-based treatment group, the mobile team group having better compliance (*p* < 0.001), as shown in Table 1.

**Table 1:** Baseline socio-demographic characteristics of respondents and their compliance (*n* = 200)

Variable	Default (Non-compliance)		*P* value
	Yes n (%)	No n (%)	
Gender			
Male	19 (14.2)	115 (85.8)	0.306
Female	6 (9.1)	60 (90.9)	
Age group			
Under 25	3 (5.9)	48 (94.1)	0.009
25 to 50	13 (11.1)	104 (88.9)	
More than 50	9 (28.1)	23 (71.9)	
Educational status			
Till primary or less	21 (15.1)	118 (84.9)	0.092
More than primary	4 (6.6)	57 (93.4)	
Nationality			
Saudi	3 (5.2)	55 (94.8)	0.045
Non-Saudi	22 (15.5)	120 (84.5)	
TB site			
Pulmonary	20 (12.4)	141 (87.6)	0.946
Extra pulmonary	5 (12.8)	34 (87.2)	
Treatment group			
Served by Mobile teams	3 (3.0)	97 (97.0)	< 0.001
Facility-based DOTS treatment	22 (22.0)	78 (78.0)	

**Fig. 1:**
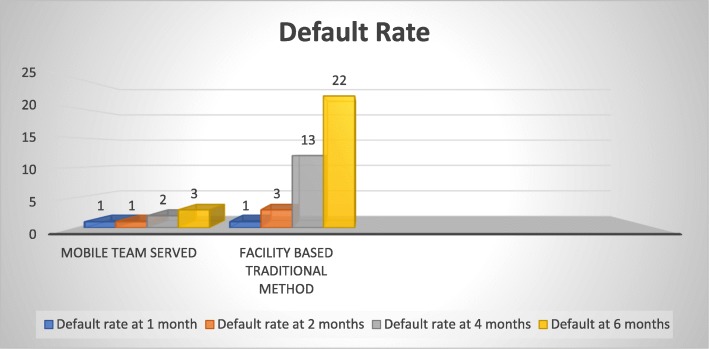
Group-wise periodic default rate

With regard to suspected sources of infection, around 77% (*n* = 152) patients were unable to identify any potential source of infection. Family members/friends and prison mates contributed to around 15.5% infection. Current cigarette smoking was reported by *n* = 16 (8%) patients and *n* = 19 (9.5%) claimed to have quit smoking. Shisha/Hukkah smoking was noted in nine (4.5%) individuals. Around *n* = 11 (5.5%) participants had a past history of TB infection (i.e. relapse), and out of these, only two (1.0%) did not complete their treatment. Periodic physical and sputum examinations were carried out at one, two, four and 6 months of treatment. Overall, the default rate was lower during the initial 2 months of the study although a sudden increase was noted in the default rate of the facility-based traditional DOTS group after 2 months of treatment, as shown in Fig. 1.

**Fig. 2:**
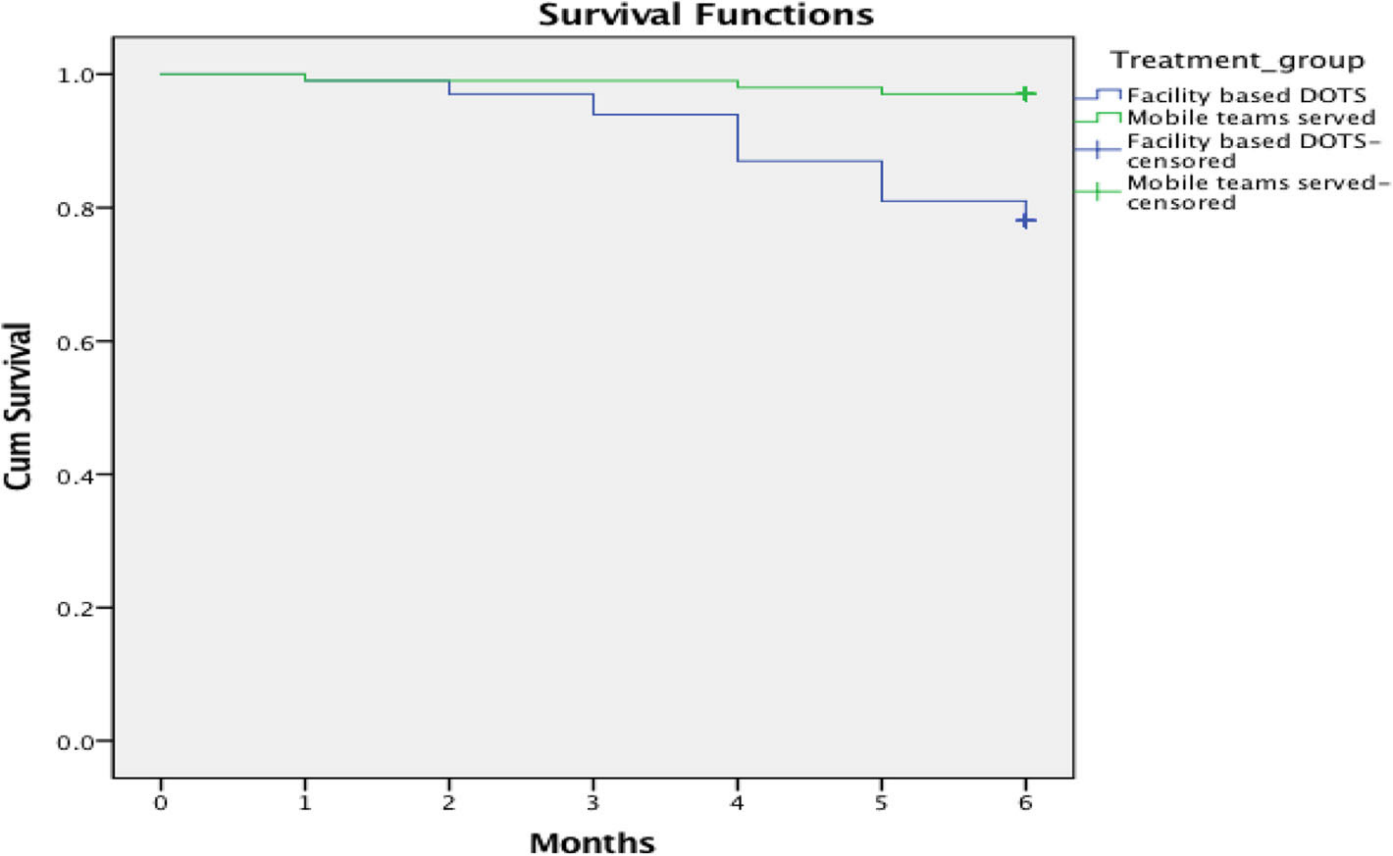
Kaplan-Meier plot of the default over time in two DOTS treatment groups

Figure 2 illustrates the Kaplan Meier analysis of default over time in the two treatment groups. In the first two to 3 months of treatment, the default rates were almost similar in both the groups. A sudden dip indicating a higher default rate in the facility-based group after 3 months of treatment was observed. There was a significant difference in the default rate of the patients in the two groups after 6 months (log rank statistics = 16.30; df = 1; *p* < 0.001).

An independent-T test identified no significant difference in the overall mean compliance score for the two treatment groups before the start of their anti-TB treatment. However, a significant difference was noted in the mean scores of those served by our mobile teams and those of the facility-based group (58.43 and 55.55 respectively) after 3 months of treatment (*p* < 0.001), as shown in Table 2.

**Table 2:** Overall mean compliance score before and after three months of treatment

Variable	TB treatment group		Mean difference	95% CI		P value
	Mobile teams served	Facility- based treatment				
	Mean (SD)	Mean (SD)		Lower	Upper	
Overall compliance perception scores						
Before treatment	45.23 (3.99)	45.28 (3.69)	0.050	−1.02	1.12	0.927
After three months	58.43 (3.46)	55.55 (3.59)	2.88	3.88	1.88	< 0.001

The patients’ ideas about the factors for non-compliance were assessed using a paired-T test for each of the groups separately, as shown in Table 3. An overall improvement in treatment compliance after 3 months was noted in both the treatment groups when compared to the baseline mean scores (*p* < 0.001 respectively). Patients in both groups agreed that having sufficient and easily-understood information about the disease would ultimately improve treatment compliance (*p* = 0.150 and *p* = 0.064 respectively). Surprisingly, after the 3 months follow-up, expressions of the fear of workplace ostracism and of social stigma about TB as possible factors affecting compliance were more prevalent in the mobile team served patients (Table 3) than in the facility-based treatment group. Other patient perception parameters for non-compliance are shown in Table 3.

**Table 3:** Patients perception for non-compliance in the two treatment groups

Variables	Community based mobile team served Mean (SD)			Traditional facility- based treatment group Mean (SD)		
	Pre *n* = 100	Post *n* = 99	P value	Pre *n* = 100	Post *n* = 96	P value
Initial rapid improvement perceived as cure	2.93 (1.04)	4.05 (0.71)	< 0.001	3.01 (0.91)	4.00 (0.76)	< 0.001
Do not feel better with TB symptoms	2.82 (0.93)	3.77 (0.81)	< 0.001	2.67 (0.93)	3.60 (0.90)	< 0.001
Forget to take medicine on time	3.44 (1.09)	4.86 (0.36)	< 0.001	3.47 (1.02)	4.33 (0.92)	< 0.001
Sufficient information about the disease, its treatment and side effects will make us more organized	4.39 (0.80)	4.47 (0.70)	0.150	4.29 (0.80)	4.41 (0.73)	0.064
Worried about the chronic diseases associated with TB	3.04 (0.86)	4.16 (0.84)	< 0.001	3.13 (0.86)	4.19 (0.78)	< 0.001
On regular treatment for other chronic diseases and drug interactions	3.28 (0.81)	4.57 (0.57)	< 0.001	3.55 (0.71)	4.42 (0.70)	< 0.001
Afraid of the treatment side effects	3.08 (1.09)	4.32 (0.75)	< 0.001	3.33 (0.98)	4.04 (0.91)	< 0.001
Fear of relapse even after full treatment course	3.22 (0.85)	4.64 (0.66)	< 0.001	3.47 (1.01)	4.62 (0.61)	< 0.001
Residence too far from health facility	3.34 (0.98)	4.90 (0.32)	< 0.001	3.20 (0.87)	4.10 (0.85)	< 0.001
Difficult to seek periodic permission from workplace to get medicine	3.46 (0.75)	4.79 (0.68)	< 0.001	3.32 (0.77)	4.28 (0.84)	< 0.001
Fear of professional (workplace) stigma	3.97 (0.86)	3.65 (0.81)	0.016	3.85 (0.93)	3.91 (0.91)	0.542
Fear of social stigma	3.64 (0.92)	3.10 (1.03)	0.435	3.61 (0.83)	3.92 (0.97)	0.009
Frequent travelling outside city/country	3.10 (1.03)	5.0 (0.0)	< 0.001	3.29 (1.14)	4.32 (0.89)	< 0.001
Females being more dependent on guardians (*n* = 36 and *n* = 30 respectively)	3.88 (0.85)	4.97 (0.16)	< 0.001	3.63 (0.80)	4.20 (0.76)	0.001
Total mean scores	45.19 (3.99)	58.43 (3.46)	< 0.001	45.35 (3.73)	55.55 (3.59)	< 0.001

Chi square analysis shows that the patients under mobile team supervision believed this treatment approach worked for them, even overcoming the problem of drug interruption/ termination on public holidays (*p* < 0.001). Similarly, regular follow up visits by the mobile teams helped to resolve patients’ queries or problems (*p* < 0.001). The mobile team served patients were generally highly satisfied with the approach, attitude and competence of the team members. The majority (93%) were willing to recommend this program to a friend or family member in future. All the study participants in both the groups were satisfied with the health education sessions.

## Discussion

The survey measured and compared patients’ compliance with TB treatment in each of the two treatment groups. The recent introduction of a new treatment strategy by the MOH involved treating TB patients by means of mobile teams who administer their medication. This new treatment strategy was intended to reduce the default rate and improve patient treatment outcomes by providing medicines to patients at the time and place of their convenience. The results of our study indicated that the community mobile outreach team approach was an effective strategy, with high acceptability amongst patients and a reduction of the default rate to only 3%,. The findings of this study also allowed us to identify and compare patients’ views and attitudes regarding non-compliance before the start of treatment, and then after 3 months of anti-TB treatment. For instance, after 3 months of treatment, significant positive changes in the ways in which the TB patients served by the mobile treatment teams viewed their mode of treatment were documented.

The high compliance rate of 97% in our mobile team served patient group was even better than the 92, 90 and 83% successes achieved in Riyadh (KSA) [5], Myanmar [32] and Pakistan [33] respectively. This significant change can be attributed to the mobile team served group’s more convenient way of receiving their medicines. It was much more difficult for patients in this group to avoid taking their medicines, as, in accordance with DOTS recommendations, they were mostly under the direct supervision of members of a mobile team [4, 33]. Another possible explanation for the overall improvement in compliance can be attributed to a higher rate of patient satisfaction with the mobile outreach approach. The high success rate appears to justify its incorporation into future NTCPP activities. Although the compliance rate was lower (around 78%) in the facility-based treatment group, it was still in accordance with the 77% of a study conducted by Ali et al. [9], and much better than the 60–69% mentioned in some South African and Indian studies [34–37].

There is still room for improvement. In some developed countries like the United Kingdom [38] and the USA [39], the reported traditional DOTS treatment success rate was around 92 and 87.2% respectively. Developed countries’ improved health care systems, coupled with better patient health education, their understanding of the associated complications of the disease and the benefits of completing the full treatment course, have all been shown to play a significant role in improving treatment compliance [38, 39].

Our results indicated that non-Saudi men (usually originating from high TB endemic countries) were not only likely to be major sources of infection, but also to have a high default rates. Abouzeid [19, 20], Alzohairy [40] and Heldel et al. [41] all recoded similar findings for non-Saudi TB patients in the KSA. Smoking (cigarette/shisha), poor living conditions, bad nutrition, and inadequate ventilation are all major disease contributors, and these sufferers are frequently to be found living like this. In addition, many of these immigrants, particularly those who are illegal, seldom visit any government health care facility for fear of being deported or imprisoned [19, 20, 40, 41]. Understanding bigoted social attitudes and the many poverty-related issues faced by this cornered and mostly uneducated community engaged in a constant struggle to eke out a living is vital in planning their treatment regimes. Acknowledging the role these issues play can assist in improved future planning, and could lead to better implementation of TB control and preventive measures [40, 41]. Periodic screening of the population, especially of high-risk groups, should improve the case detection rate. With prompt intervention, taking into consideration the patient’s own situation and the simplification of drug administration regimes, like that of the mobile team approach, limiting disease transmission and increasing cure rates will become possible.

The literature shows that the long duration of self-administered drug therapy together with the need for regular health center visits to obtain new drug supplies are generally the main hurdles to be overcome when persuading patients to continue treatment [28, 29]. We know that drug interruption results in relapse and multidrug resistance [30, 31]. The NTCPP and the health planners of the MOH have tried to overcome this obstacle, and we see the introduction of the mobile team approach as an important step forward [5]. Strong evidence for this is provided by the relatively high patient default rate in the facility-based treatment group in our study, as compared to the mobile team served cohort. The long treatment period, early symptom relief, the fear of drug adverse effects, associated chronic diseases in sufferers, and the social stigma attached to TB were all shown to be important factors leading to the interruption of treatment. These reasons for default were also cited by Ifebunandu et al. [29] and Sanchez et al. [30]. The improvement in the scores of almost every compliance perception parameter shown in our study, particularly so in the mobile team outreach group, clearly showed how effective this approach can be. The findings of our study also highlighted the significance of quality health education, as well as the need for trust and good communication between the mobile team members and their patients [5].

Our results showed that a substantial proportion of patients in the facility-based DOTS group defaulted during the continuation phase of treatment (i.e., after the initial 2 months). The results were consistent with the findings of a systemic review on the timing of TB treatment default [42]. Here, long treatment duration and early symptom relief were perceived as main contributory factors. The deportation of illegal residents before treatment completion could also be another possible reason [16–19, 38, 41]. However, these findings contrast with the those of studies conducted in Nigeria [29], Kenya [6], Brazil [11] and Uzbekistan [31], where the default was noted more during the intensive phase (first 2 months) of treatment. Possible explanations identified by these studies included: poor information about the disease, early symptom relief erroneously making defaulters believe that they had been cured, a high economic burden (the need for travel, the drug price itself, and long waiting times) for each visit, side effects of drugs, and related co-morbidities. Our study results indicated that those patients having no or minimal education and those in an older age group were more likely to default. Similar findings were reported by Mohammad et al. [33].

Previous studies report more non-compliance in female patients, attributing it to females’ dependence on their male partners to attend the clinic [1, 5, 12]. Often, social and religious conventions forbid females to travel alone. To overcome and address this particular obstacle, the mobile team initiative proved to be a considerable success. Similarly, another argument for non-compliance put forth by some researchers is poor public transport or lack of availability of any transport whatever, especially for those living far from health care centers [11, 12]. This concern, too, was addressed by our mobile team initiative. Chhaya et al. [28] in their study identified more defaulters in male patients, attributing it to the difficulty of taking frequent leave of absence from the workplace. Study participants served by our mobile team did not have to worry about such restrictions, resulting in better compliance.

In contrast to the above discussion, some previous studies have reported that patients holding regular jobs were less likely to default as a result of their higher socio-economic and educational status, and the assumption is that they have a better understanding of the drug regimen and a better understanding of the consequences of not completing the course [29, 33]. The dedication and commitment of our TB control program staff resulted in the improved understanding scores relating to drug side effects and the importance of completing the full course of treatment amongst our study participants. They also helped to dispel their patients’ misconceptions about relapse and their fear of associated diseases. Participants in both the cohorts initially anticipated that seeking periodic permission for absence from their workplace to get medicine would be difficult. However, when asked about this after 3 months of treatment, it appeared that this was not really such a major concern for our study participants. Although compliance and satisfaction with the mode of treatment was high in the mobile outreach team patients, after 3 months a surprisingly high number of them were still more concerned about the social stigma and bigotry associated with TB. Periodic visits by at least two mobile team staff members at a time at a workplace or even a residence can draw attention to these patients in the community or their workplace. This we believe is a major drawback of the mobile team approach, and one which needs to be looked into.

The study had a few limitations. Budgeting for the transportation of the mobile teams needed to be determined in advance, and the availability of transport when it was required was not always considered. Another potential limitation for this study was the difficulty in active case finding by screening and contact tracing, since the meeting point for a few patients was not their houses. Difficulties like strange or aggressive conduct on the part of the patients or their relatives, and problems faced by female members of the mobile team members were also not assessed. The seeking of traditional remedies to treat TB by potential subjects was also not examined.

## Conclusion

In conclusion, this study showed our mobile outreach DOTS approach to be an effective and acceptable strategy for the ongoing treatment of TB patients. Lower default rates among patients and improvements in patients’ understanding of the disease highlighted the positive and significant impact of our mobile outreach teams. In the fight against the increased burden on the state posed by TB, with its associated costs and health risks to other members of the population, not only in our region, but all over the Kingdom of Saudi Arabia itself, the need for a new strategy is imperative. Since the effectiveness of the mobile team approach has been demonstrated by improved patient knowledge of the disease and a significant decrease in the treatment default rate, the adoption of this concept for all TB patients in Jeddah and its replication in other cities of the KSA is thus recommended.

## Data Availability

All the data is available with Infection control department of MOH. Data are available on request due to privacy or other restrictions.

## Abbreviations

AFB: Acid Fast Bacilli
DOTS: Directly Observed Therapy Short course
KSA: Kingdom of Saudi Arabia
MOH: Ministry of Health
NTCPP: National TB Control and Prevention Program
PHCC: Primary Health Care Center
TB: Tuberculosis
WHO: World Health Organization
